# MEF2 signaling and human diseases

**DOI:** 10.18632/oncotarget.22899

**Published:** 2017-12-04

**Authors:** Xiao Chen, Bing Gao, Murugavel Ponnusamy, Zhijuan Lin, Jia Liu

**Affiliations:** ^1^ School of Pharmacy, Qingdao University, Qingdao 266021, China; ^2^ Institute for Translational Medicine, Qingdao University, Qingdao 266021, China; ^3^ School of Basic Medicine, Qingdao University, Qingdao 266021, China

**Keywords:** MEF2, human diseases, signaling pathway, microRNAs

## Abstract

The members of myocyte Enhancer Factor 2 (MEF2) protein family was previously believed to function in the development of heart and muscle. Recent reports indicate that they are also closely associated with development and progression of many human diseases. Although their role in cancer biology is well established, the molecular mechanisms underlying their action is yet largely unknown. MEF2 family is closely associated with various signaling pathways, including Ca^2+^ signaling, MAP kinase signaling, Wnt signaling, PI3K/Akt signaling, etc. microRNAs also contribute to regulate the activities of MEF2. In this review, we summarize the known molecular mechanism by which MEF2 family contribute to human diseases.

## INTRODUCTION

MEF2 transcription factor plays vital role in both physiological and pathological processes. The members of MEF2 family have been mainly involved in neural development, muscle formation, heart development, and carcinogenesis. Emerging evidences indicate that MEF2 family contributes to the development of various human diseases due to its complex function by interacting with numerous signaling pathways and small non-coding RNA such as microRNAs [1–4]. This review summarizes the relationship between MEF2 family and their molecular mechanisms of development and progression of several death causing human diseases. We also described signaling pathways and microRNAs directly involved in regulation of expression and activity of MEF2. In addition, we have summarized some of the new drugs that targeting MEF2 family.

## BASIC KNOWLEDGE OF MEF2 FAMILY

### The MEF2 family and gene mapping

**Figure 1:**
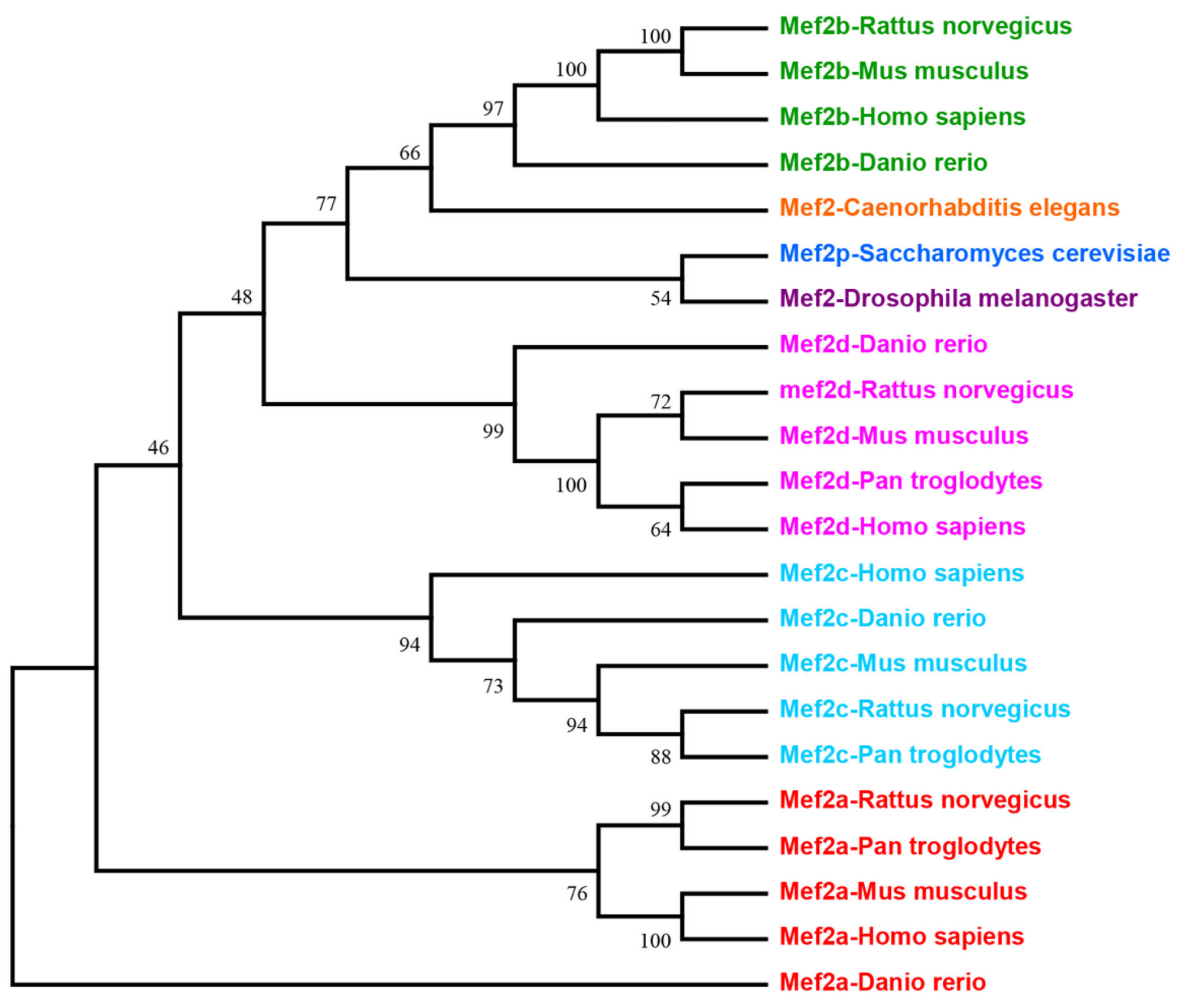
This is the phylogenetic tree of MEF2 family Evolutionary analyses tree of MEF2 family was conducted in software MEGA7 by using Neighbor-Joining method.

MEF2 proteins belong to MADS-box family of transcription factors. MADS-box is named from the four proteins minichromosome maintenance genes (MCM1), agamous (AG), deficiens (DEFA) and serum response factor (SRF), which share a contiguous conserved motif in eukaryotic organisms [5, 6]. MEF2 proteins were initially identified as a vital factor for skeletal muscle development that have the ability to bind A/T-rich sequences within gene promoter of muscle creatine kinase (MCK) [7, 8]. MEF2 is a single gene in *Drosophila*, *Caenorhabditis* elegans and *Saccharomyces cerevisiae,* while vertebrates have four distinct numbers—namely, MEF2A, B, C and D [3]. The evolutionary history of MEF2 family is inferred using the Neighbor-Joining method (Figure 1). The sole MEF2 gene in *Drosophila melanogaster* is located on chromosome 2. Murine MEF2A, C and D are located in chromosome 7, 13 and 3, respectively. A study deduced that MEF2B may be located on chromosome 8. In human, MEF2A, B, C and D are confirmed to be located on 15q26, 19q12, 5q14 and 1q12-q23, respectively [9].

### The structure of MEF2 proteins

**Figure 2:**
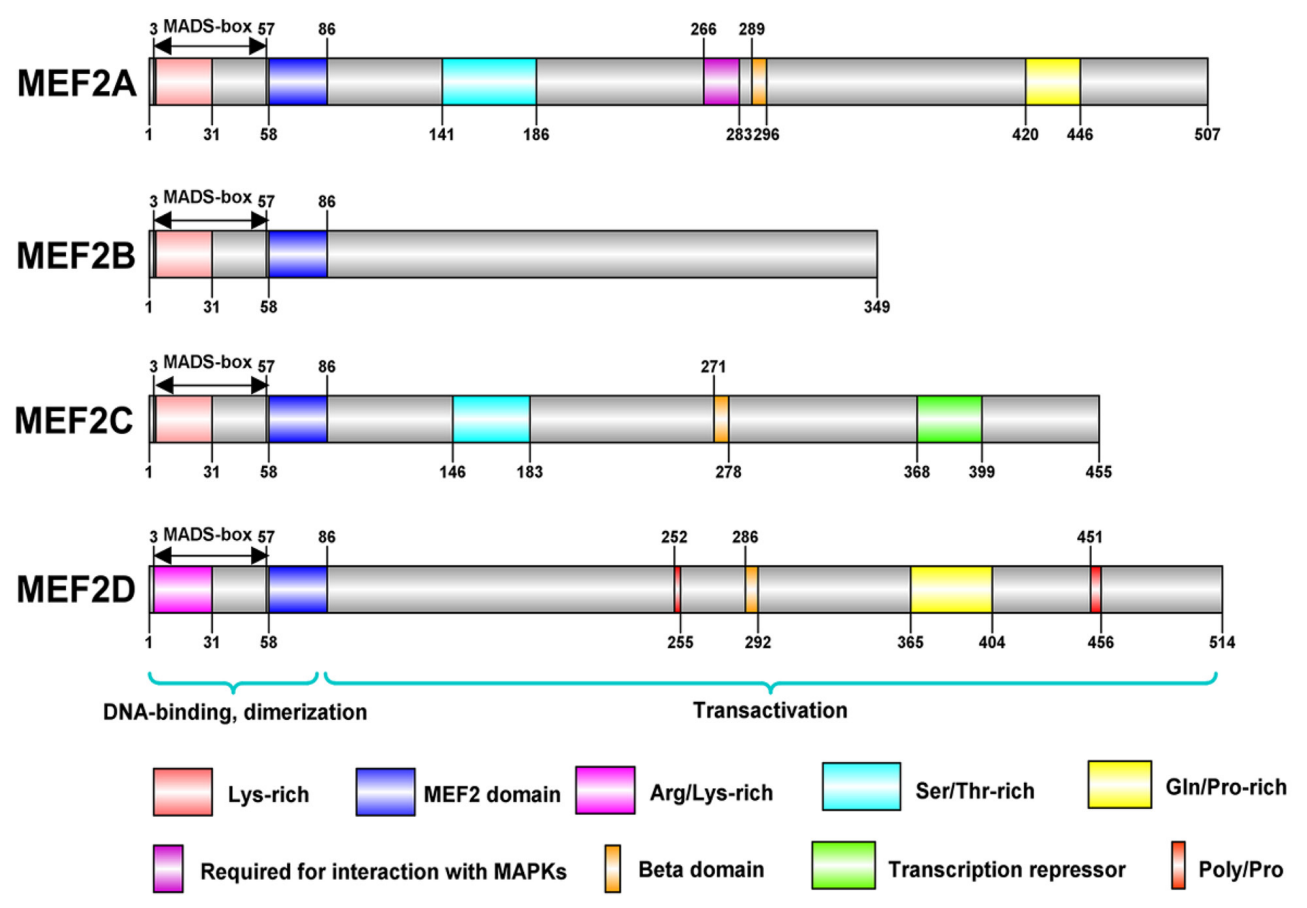
These are structures of MEF2 proteins in human The structures of MEF2 proteins were conducted in IBS(illustrator for Biological Sequences). MEF2 family proteins, including MEF2A, B, C, and D, share a similar N-terminal that contains a highly conserved MADS-box domain and an immediate adjacent MEF2 domain. MADS-box and MEF2 domain have 55 and 29 amino acids, respectively, and both contribute to DNA binding and dimerization. There is a diversity in the structure of C-terminal transactivation domain of MEF2 proteins. MEF2B is the simplest protein among MEF2 proteins. MEF2A, C, and D contain several amino acids with the potential of phosphorylation.

MEF2 family proteins from different species share a very similar N-termini that contains a highly conserved MADS-box domain and an immediate adjacent MEF2 domain, however, there is a diversity in the structure of C-terminal transactivation domain [4] (Figure 2). The MADS-box domain which is a DNA-binding region of MEF2 protein consists of 55 amino acids with some conserved residues shared by other transcription factors of MADS-box family, and this region plays a key role in the recognition of their target sequences. The main role of these invariable residues are combined with abundant A/T DNA sequences and they mediate dimerization of MADS-box proteins and provide places for interaction of MEF2 with other cofactors. Due to the conserved sequence within MEF2 domain, MEF2A, B, C and D can form homo-dimerization structures with themselves, or hetero-dimerization with other molecules [10]. In C-terminal part of MEF2 proteins, conserved regions contain potential phosphorylation sites recognized by some specific kinases, but other amino acid sequences are highly diverse. Intriguingly, alternative splicing of MEF2 mRNAs take place in the sequences coding their C-terminal regions, which contribute to the regulation of MEF2 proteins’ activity [11].

### Transcriptional targets of MEF2 family

In the neural development, MEF2 has directly engaged in many genetic programs. The previous publications had identified that the (C/T)TA(T/A)_4_TA(G/A),a ten-nucleotide motif , could be MEF2 binging site. In addition, the activity-dependent MEF2 genetic program may use polyA site switch mechanisms to inference the functions of proteins. The activity-regulated neuronal target genes of MEF2 and Poly A switch are JSAP1, Odz2, Arhgef9, Csnk1e, Vesl-1, Omp25, Pkib, Septin 11, Rinzf, Klf6 and Tcf4 [12]. In the cardiovascular development, especially during mouse embryogenesis and adulthood, myocardin had been proved to be a direct transcriptional target of MEF2,Tead and Foxo proteins [13]. Consistently, myomaxin also had been identified as the direct downstream target gene of MEF2A [14]. During the *Drosophila* embryonic development, researchers had constructed a temporal map of MEF2 activity by immune-precipitation analysis and gene expression profiles. The results indicated that Mhc, mbl, nau and meso18E were directly regulated by MEF2 [15]. Recent researches have determined the putative recognition motif of MEF2 transcription factors within the promoter or enhancer of their downstream target genes, which are enriched in the constitution of adenine and thymine. Recently, researchers used a powerful approach to illuminate the genomic functions of MEF2A and MEF2C and to identify their transcriptional targets by ChIP-seq in mouse cortical neurons. MEF2A and MEF2C may encode similar epigenetic programs by binding enhancer regulatory elements close to the target genes involved in neuronal plasticity and calcium signaling. The results indicated SRF could bind to MEF2,AP1and NeuroD1,CPBE/ATF could bind to MEF2A enhancers and CEBP/A and forkhead box factors could bind to MEF2C. And the LRP8-Reelin-Regulated Neuronal enhancers (LRN) could be recognized by both MEF2A and MEF2C in synapse-to-nucleus pathway. According to KEGG pathway analysis, the MEF2 factors could regulate signaling pathways including glutamatergic synaptic transmission, drug addiction, axon guidance and MAPK signaling pathways [6]. During the evolution of abthropoid primates, a new enhancer element emerged due to 5-10 nucleotide changes in osteocrin (OSTN). The element was an 85 bp-long sequence and located about 600 bp upstream of OSTN transcription initiation site. Intriguingly, MEF2 can bind to this sequence, indicating that OSTN is a potential target of MEF2s. Further study on the mechanism between MEF2 and OSTN may contribute to our better understanding of human cognition and brain function. [16].

## MEF2 PHYSIOLOGICAL FUNCTION AND SIGNAL PATHWAYS

**Figure 3:**
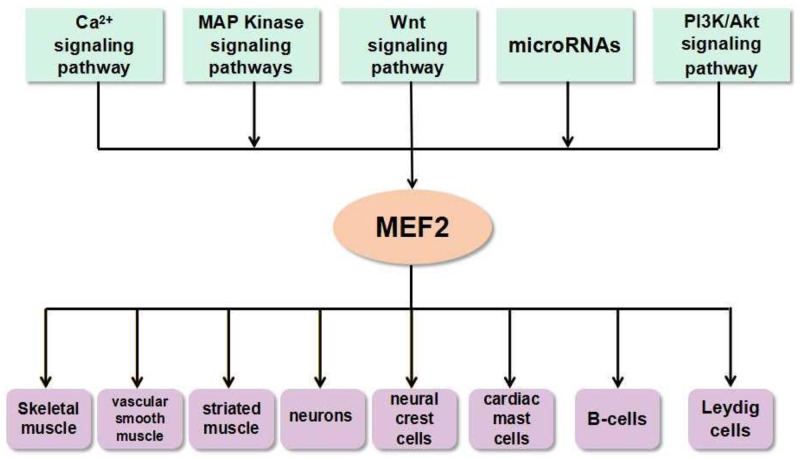
The link between MEF2 proteins and signals in various types of cells and tissues The microRNAs and signaling pathways such as Ca2+ signaling pathway, MAP Kinase signaling pathways, Wnt signaling pathway, PI3K/Akt signaling pathway, etc. can activate MEF2. This interaction between MEF2 proteins and signals ubiquitously exist in many types of cells and tissues.

MEF2 family is mainly associated with a variety of physiological processes, including muscle formation [17], nervous system development [18], heart development [19], etc. They are also implicated in human diseases, such as liver fibrosis [20], cancers [21] and neurodegenerative diseases [22]. Atkins et al. established a Ras-driven tumorigenesis model in Drosophila epithelial tissues to study gene regulatory networks in cancer cells. They discovered that Mef2 is involved in a cross-regulation with other factors such as Sat, AP-1, Myc, AP-4, Ftz-f1, and Taiman/SRC3 [23]. MEF2 family can be regulated by multiple signaling pathways during normal physiological processes as well as in pathological conditions (Figure 3). Accumulating evidence show that MEF2 proteins promote the differentiation of skeletal, cardiac and smooth muscle myocytes [24, 25]. MEF2 proteins are widely expressed in muscle and nervous tissues, and they can integrate extracellular signaling into promoters or enhancers of tissue-specific genes. Some researchers revealed a link between MEF2A and SRF in the activation of selective muscle-specific promoters in different muscle cell lines. Similarly, MEF2C and SRF can cooperatively regulate the expression of miR-133a [26]. MEF2 activity is controlled by the myogenic regulatory factor MRF4 in the adult skeletal muscle [27]. A recent study showed a significant up-regulation of MEF2C and MEF2D mRNA levels in both sporadic and SOD1^+^ amyotrophic lateral sclerosis (ALS) patients detected by gene expression analysis, and a down-regulation of their targets, BDNF, KLF6, and RUFY3 [28].

The family members of MEF2 transcription factor are closely associated with calcium-dependent signaling pathway [8], which play an important role in the development of nervous system and neuronal differentiation [29]. All MEF2 family members, especially MEF2C, are highly expressed in the neuron of central nervous system [30]. MEF2A and MEF2D are found to exhibit transcriptional activity in post-mitotic cerebellar granule neurons [31]. Surprisingly, MEF2A, C, and D play a distinct role in cell- and non-cell-autonomous control of adult hippocampal neurogenesis [32]. MEF2B acts as a valuable marker of normal germinal center (GC) B cells, and it is potentially useful for differential diagnosis of small B cell lymphomas [33]. As an Epstein-Barr virus (EBV) nuclear antigen 1 (EBNA1)-bound gene, MEF2B is important for the survival of B cells infected with EBV [34].

As a cardiac-specific marker gene, MEF2 can be regulated by focal adhesion kinase to enhance the expression of Jun in cardiomyocytes [35]. MEF2C is also associated with congenital human heart defects due to its regulatory function on teratocarcinoma-derived growth factor 1 expression (Tdgf1), which is essential for the early embryonic heart development. The abnormalities in the expression of MEF2C and its associated Tdgf1 leads to developmental defects and congenital heart problems [36]. Mef2c can physically interact with Suv39h1 and their interaction contributes to the regulation of myogenic expression, histone methylation modification, and myoblast differentiation. Furthermore, MEF2 transcription activity could be inhibited by Suv39h1 in a dose-dependent manner [37].

MEF2 family members also have close connections with biological characteristics (e.g., uncontrolled proliferation and enhancement of invasion) and clinical outcomes of cancer. MEF2D is overexpressed in colorectal cancer tissues and it promotes cancer cell invasion and metastasis [38]. A study in our laboratory also found that MEF2D acts as an oncogene in HCC. The expression of MEF2D is up-regulated in hepatocellular carcinoma (HCC) and this increase can accelerate cell proliferation in HCC [39]. MEF2D co-operationally promotes HCC invasion with other oncogene, such as Pokemon [40]. Molecular studies revealed that MEF2 family members promote invasion properties of HCC by enhancing the effect of TGF-β1 on EMT [41]. Increasing evidences indicate that MEF2C and MEF2D both act as tumor-promoting or -suppressing proteins dependent on the type of cancer. In leukemic patients, the ectopic expression of MEF2s caused by chromosomal rearrangements is responsible for the development of leukemia. A high level expression of MEF2C is observed in T-cell acute lymphoblastic leukemia (T-ALL) [42, 43]. The chromosomal translocation generates reciprocal DAZAP1/MEF2D and MEF2D/DAZAP1 fusion genes that promotes oncogenic properties in NIH 3T3 cells [44]. In contrast, the depression of MEF2C and MEF2D can promote cell proliferation and anchorage independent growth in lipo- and leiomyosarcoma by upregulating HDAC4 and PI3K/Akt signaling [45].

### The involvement of MEF2 family in Ca^2+^ signaling pathway

MEF2 family often acts as an effector of many extracellular and intracellular signals. Ca^2+^ signaling pathway is the major regulator of MEF2 activity by multiple mechanisms. MEF2 is required for calcineurin signaling in developing skeletal muscle [46]. MEF2 mediates calcium/calmodulin-dependent signaling responsible for the terminal differentiation of skeletal muscle progenitor cells. In specifically, the activation of transcriptional activities of MEF2 proteins by calcineurin, a calcium/calmodulin-dependent protein phosphatase, is required for myogenic differentiation [47]. A further study showed that scaffolding protein mAKAP organizes a calcineurin/MEF2 signaling complex in myocytes, and which regulates gene transcription promoted by MEF2 family members [48].

Apart from this, several cofactors and kinases also regulate MEF2 activity in calcium signaling. Hu et al. (2015) found that endothelin signaling activates various gene expression in neural crest by enhancing MEF2C activity through Calmodulin-CamKII-histone deacetylase signaling cascade [49]. In addition, salt inducible kinase 2 (SIK2) tightly regulates CaMKI dependent transcriptional activities of MEF2C and disruption of SIK2 promotes activation of CaMKI depdendent activation of MEF2C mediated gene expression during cell proliferation and stress response [50]. Interestingly, a novel MHCI signaling regulates MEF2 transcription factors. MHCI pathway requires calcineurin-mediated activation of MEF2 to control synapse density in young rat cortical neurons [51]. In addition, Tsai1 *et al.* found that the activation of MEF2 proteins could induce synapse elimination and degradation of postsynaptic density protein 95 (PSD-95) through protein phosphatase 2A (PP2A)-mediated dephosphorylation of murine double-2 (MDM2) in wild-type neurons. However, eukaryotic elongation factor 1α (EEF1a) could inhibit MEF2-induced accumulation of MDM2 at the synapses in Fmr1 KO mouse neurons [52].

Surprisingly, MEF2 is not only expressed in myocytes and neurons, but also detected in Sertoli and Leydig cells of fetal and adult testis. In MA-10 Leydig cells, steroid hormone biosynthesis and steroidogenic gene expression are regulated by luteinizing hormone (LH), which activates Ca^2+^ signaling pathways and MEF2 transcription factors [53]. Another study showed that MEF2 itself can bind and activate Gsta1 promoter as well as cooperate with Ca^2+^/calmodulin-CamKI to further enhance the initiation of Gsta1 transcription in Sertoli and Leydig cells [54].

### The function of MEF2 proteins in MAP kinase signaling pathways

The mitogen-activated protein kinase (MAPK) signaling pathways are ubiquitous type of serine/threonine protein kinase, which plays an important role in many biological processes (cell proliferation, cell differentiation, cell apoptosis, and etc.) [55, 56]. There are three independent MAPKs signaling pathways such as extracellular signal-regulated kinase (ERK) pathway, c-Jun N-terminal kinase/stress-activated protein kinase (JNK/SAPK) pathway and p38 MAPK pathway are found in mammalian cells [57], and MEF2 factors have been frequently found to be activated by these signaling pathways.

ERK protein kinase comprises five members namley ERK1, ERK2, ERK3, ERK4 and ERK5. Among them, ERK5 is closely associated with the activation of MEF2 transcription factors. In myeloid leukemia cells, ERK5 promotes activation of its direct downstream target, MEF2C transcription factor, and this activation required for the monocytic differentiation of leukemic cells [58]. In sensory neurons, the ERK5/MEF2D pathway strictly regulates expression of Bcl-w, an anti-apoptopic bcl-2 family member, which promotes sensory neuron survival [59]. Recently, a groundbreaking finding revealed that the activation of MEF2C/D transcription factors promote the development of early B-cell, which depends on their phosphorylation by ERK5. These researchers also reported that B-cell development is blocked at the pre-B-cell stage in MEF2C/D-knockout mice [60]. The ERK5-MEF2C signaling also plays a critical role in anti-apoptotic and neuroprotective actions of ischemic preconditioning in the hippocampus CA1 region [61]. In Hela cells, ERK5/MEF2B signaling can upregulate apoptosis suppressor induced by DNA damage and promotes cancer cell invasion by activating β-catenin target genes [62].

p38 is an important member of MAPK family, which can be activated by physiological stress, lipopolysaccharide, osmotic stress and ultraviolet irradiation [63]. The relationship between p38 MAPK and MEF2s was first identified in myocardial cells. p38 MAPK can activate MEF2C by phosphorylation of three amino acids located in C-terminal active region [64]. p38 MAPK-mediated phosphorylation-dependent activation of MEF2s increases its interaction with β-catenin, which stimulates cell proliferation in multiple cell types including primary VSMCs [65]. More intriguingly, MEF2 proteins are found to negatively regulate p38 MAPK pathway in a feedback fashion [66]. In recent years, more evidence have been shown that p38 MAPK pathway activates the expression of MEF2 genes under a variety of cellular circumstances. For instances, p38 MAPK signaling activates MEF2C to control B cell differentiation [67] and this signaling axis also promotes osteogenic differentiation [68]. In myocardial hypertrophy, MEF2 family is associated with increased heart mass in response to pressure overload, and its activation in hypertrophic heart requires adiponectin signaling mediated upregulation of p38 MAPK pathway [69]. Some researchers identified that microRNAs are critically regulating MEF2 and p38 MAPK axis. For example, miR-140 is a suppressor of p38 MAPK signal transduction pathway, and overexpression of this microRNA can reduce MEF2C expression [70]. Glycogen Synthase Kinase 3β (GSK3β) is a well-known regulator of striated muscle-related gene expression, which suppresses both myogenesis and cardiomyocyte hypertrophy. It is well known that MEF2 transcription factors are essential for the regulation of skeletal and cardiac muscle gene expression. GSK3β can regulate MEF2 activity indirectly through regulation of p38 MAPK [71].

### The link between MEF2 and Wnt signaling

The Wnt signaling is a kind of evolutionarily conservative signaling pathway that exists widely in invertebrates and vertebrates. The Wnt signaling plays a vital role in developmental and physiological processes such as animal embryonic development [72], organ formation and tissue regeneration [73]. The Canonical Wnt/β-catenin pathway can activate expression of many nuclear target genes, and the aberrant activation of the Wnt signaling is closely implicated in malignant transformation and progression [74, 75] and blocking of Wnt/β-catenin signaling inhibits cancer cell proliferation. While a very high expression of MEF2C leads to risk of VEGF-induced malignancy [76].

The proteins of MEF2 family also serve as downstream targets of Wnt/β-cateinin pathway genes, and mediate its physiological functions. MEF2A and Wnt signaling both contribute to skeletal muscle regeneration in adult mice. A molecular study found that MEF2A promotes this process via directly regulating two microRNAs, miR-410 and miR-433. In MEF2A knockout mice, these two microRNAs are downregulated during skeletal muscle regeneration, which results in upregulation of SFRP2 expression and reduction of Wnt activity [77].

### microRNAs associated regulation of MEF2 proteins

**Figure 4:**
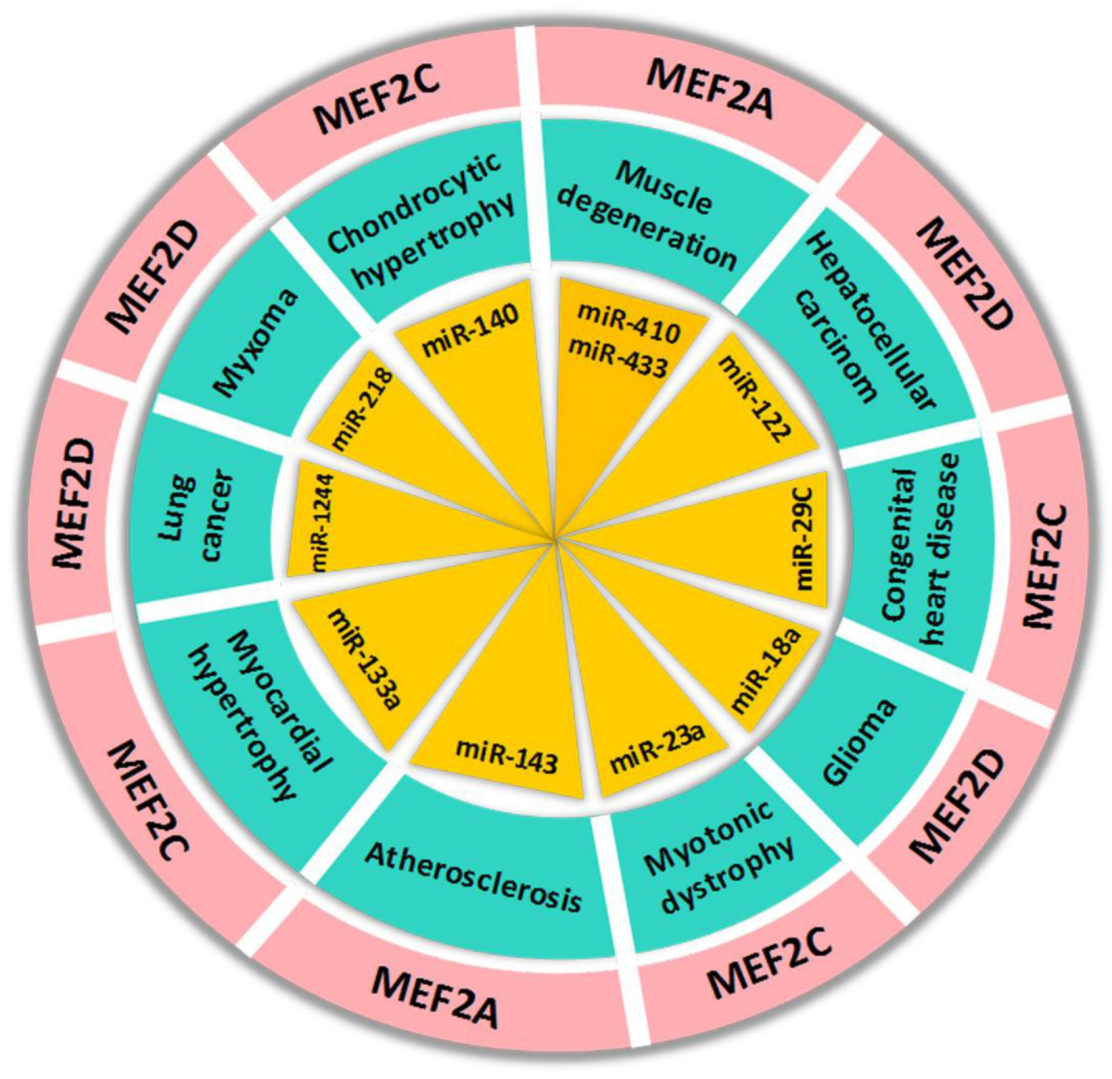
The regulation of MEF2 proteins by microRNAs in human diseases The microRNAs in the figure can regulate MEF2 expression by targeting 3’UTR of its mRNA in human diseases.

Many recent studies observed that microRNAs, a type of small non-coding RNAs, participate in the regulation of MEF2s, especially in the progression of malignant diseases. miR-218 regulates MEF2D expression by targeting 3’UTR of its mRNA and down-regulation of miR-218 in cardiac myxoma condition can increase MEF2D expression and promote cell cycle progression [78]. In glioma cells, miR-18a binds with 3'UTR of MEF2D mRNA and negatively regulates its expression [79]. Similarly, miR-122 and miR-1244 negatively regulate MEF2D expression in hepatocellular carcinoma and lung cancer cells, respectively [39, 80]. Furthermore, MEF2D regulate miR-1244 by directly binding to its promoter and this molecular regulatory loop could be a target for lung carcinoma treatment [80]. Apart from their role in cancer, the regulatory functions of MEF2s are also well established in other human diseases. MEF2C is the cardiac-specific markers and its expression is upregulated in congenital heart disease (CHD) due to downregulation of the expression of miR-29C [81]. Li et al. (2015) found that MEF2D is an authentic target of miR-103, which promotes cell differentiation by targeting the AKT/mTOR signaling mediated activation of MEF2D [82]. In hyperhomocysteinemia induced cardiac hypertrophy, the downregulation and inactivation of MEF2C leads to attenuation of expression of miR-133a, an anti-hypertrophic factor in cardiomyocytes. Hydrogen sulfide (H2S) can mitigate hypertrophy via reversal of expression of miR-133a through activation of MEF2C in hyperhomocysteinemia cardiomyocytes [83]. Similarly, MEF2A and MEF2C regulate miR-143 and miR-23a activities via their interaction with 3’UTR region of microRNA in vascular smooth muscle cell (VSMC) and cardiomyocytes from mice with myotonic dystrophy, respectively [84, 85]. These studies reveal that microRNA/MEF2 pathway might play a key role in various types of cells and its dysregulation always contribute to various diseases (Figure 4). Therefore, targeting this molecular pathway could be a promising therapeutic strategy.

Besides, some studies have revealed that long noncoding RNAs (lncRNAs) play important roles in regulating some critical transcription factors, including MEF2A-D. Linc-MD1 is a kind of competing endogenous lncRNAs that its reduced expression level in Duchenne muscular dystrophy (DMD) promotes differentiation of myoblast. Notably, linc-MD1 detaches miR-135 away from MEF2C mRNA, and thus, depresses the expression of MEF2C in skeletal muscle development and disease [86]. Song and his/her colleagues found that the expression level of uc.167 was robustly elevated in ventricular septum defect (VSD) heart tissues and uc.167 had an effect on cell proliferation, apoptosis and differentiation of P19 cell by regulating MEF2C. In addition, the overexpression of uc.167 induced the reduction of MEF2C expression. Altogether, the concrete relationship between MEF2C and uc.167 still needs further investigation [87].

### Other signaling pathways associated with MEF2s

The PI3K/Akt pathway is a newly confirmed MEF2-associated pathway, which is involved in the proliferation, differentiation, apoptosis and the regulation of glucose transport. The PI3K/Akt pathway is closely implicated in the occurrence of many human diseases [88, 89]. The PI3K/Akt signaling can enhance MEF2 transcriptional activity in muscle differentiation [90]. Interestingly, the depletion of MEF2D promotes neonatal cardiomyocyte proliferation by suppressing the expression of PTEN, which is the primary negative regulator of PI3K/Akt signaling. However, prolonged depletion of MEF2D in neonatal cardiomyocytes resulted in significant programmed cell death. This study suggest that MEF2D mediated regulation of PI3K/AKT signaling contributes to post-mitotic state of cardiomyocytes [91]. In contrast to this, a molecular study in sarcoma cells found that PI3K/Akt pathway influences the expression of target genes of MEF2-HDAC axis and increased activity of PI3K/Akt results in decreased expression of MEF2 [45].

Cyclic-AMP dependent protein kinase A (PKA) is a kind of protein kinase with simple structure, which is also connected to the transcriptional activities of MEF2. MEF2 can be a target of cAMP-PKA pathway in neuron [92]. Given the fact that MEF2 proteins are key regulators myogenesis, cAMP-PKA-pathway-mediated myogenic repression occurs through downregulation of MEF2D in myoblasts and this loss leads to inhibition of the skeletal muscle differentiation program. Given the fact that MEF2 proteins are key regulators of myogenesis, cAMP-PKA-pathway-mediated myogenic repression occurs through downregulation of MEF2D in myoblasts and this loss leads to inhibition of the skeletal muscle differentiation program [93].

In iron/sphingolipid/PDK1/Mef2 pathway, loss of FXN in vertebrates can upregulate 3-phosphoinositide dependent protein kinase-1 (Pdk1) and myocyte enhancer factor-2 (Mef2), and induce sphingolipid synthesis. Furthermore, the activation of Mef2 triggers the aberrant transcription of downstream targets. Similarly, loss of frataxin homolog (fh) in Drosophila nervous system causes the accumulation of iron, and in turn, enhances spingolipid synthesis. In addition, PDK1 and Mef2 were both activated to trigger neuro-degeneration of adult photoreceptors. Since the above evidence has well documented that the mechanism of iron/sphingolipid/PDK1/Mef2 pathway is conserved among different species, targeting this pathway may benefit on the hearts of Friedreich ataxia (FRDA) patients [94].

As mentioned above, MEF2 is a single gene in *Drosophila*, and it is a vital gene in myogenesis of indirect flight muscles (IFMs). MEF2 can inhibit Notch pathway in non-myogenic cells [95]. Like other molecular regulator, MEF2s can also cooperate with key proteins in other signaling to modulate its final biological effect. For example, the Notch-MEF2 synergy acts upon JNK signaling and its downstream events to induce proliferation and metastasis in *Drosophila* [96].

## DISCUSSION

Researchers are engaged in developing new drugs that target MEF2 family in order to treat MEF2 associated human diseases. For instances, oleanolic acid is a new type of anti-tumor drugs that suppresses proliferation of lung cancer cells via inhibition of MEF2D expression [97]. Similarly, estrogen alone or its co-treatment with parathyroid hormone can increase vertebral bone mass by suppressing the expression of MEF2s in ovariectomized rats [98]. Atorvastatin is a drug used to treat angiocardiopathy, which can reverse cardiac remodeling by suppressing protein kinase D/MEF2D activation in spontaneously hypertensive rats [99].

**Figure 5:**
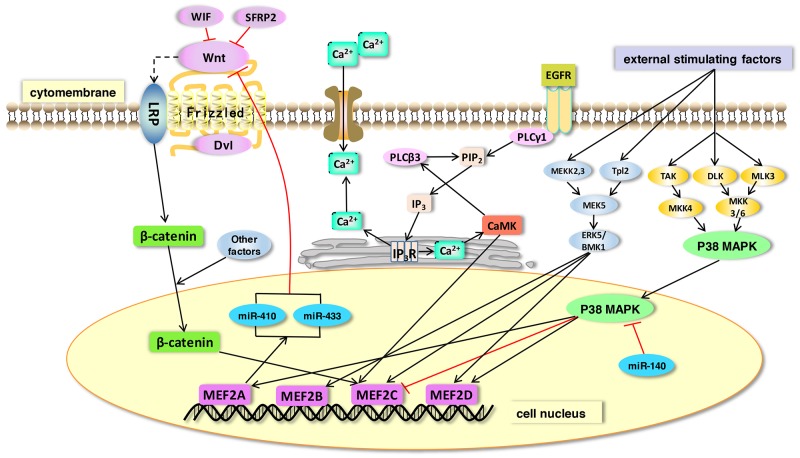
The function of MEF2 proteins in signaling pathways MEF2 proteins act as effectors of many signaling pathways. In this figure, we show some representative pathways. 1) Wnt protein binds frizzled receptors to activate its downstream reactions. A stablized β-catenin is then translocated into the nucleus where it binds with MEF2 transcription factors. MEF2A can directly regulate microRNAs (miR-410 and miR-433) and thus inhibit the Wnt signaling pathway. 2) MAPK signaling (ERK and p38 MAPK) can be stimulated by external factors. The ERK signaling increases expression of its direct downstream targets, MEF2B, C and D. MEF2A and MEF2C can be activated by p38 MAPK. miR-140 is a suppressor of p38 MAPK signaling and its overexpression can decrease MEF2C expression in a p38MAPK-dependent pathway. 3) The expression of MEF2C can be enhanced by the activation of CaMK in Ca2+ signaling pathway.

In this review, we summarized that MEF2 family plays significant roles in nervous system diseases, heart diseases, muscular disorders, and cancers (Table 1). The four members in MEF2 family are actually different from each other in their biochemical and physiological features, and these distinctions cause their different roles in human diseases. Although many signaling have been identified as a partner of MEF2 proteins, more new molecular pathways may be related to these transcription factors. MEF2s may participate in the regulation of YAP/Hippo signaling and EGFR-related pathway. Similarly, NF-kappa B pathway may also be regulated by MEF2 proteins during inflammatory reactions. Taken together, MEF2 proteins and their associated signaling pathways play important roles in both physiological and pathological process in human (Figure 5).

**Table 1:** The MEF2-related human diseases, signaling pathways, targets and drugs

Diseases	Mainly corresponding MEF2 subtype	Signaling Pathways	Targeted Proteins	Drugs
Nervous system diseases	MEF2A MEF2C MEF2D	MAP Kinase signaling pathway P38 MAPK pathway Extracellular signal-regulated kinase (ERK) pathway Ca^2+^signaling pathway	SIK2,PSD95, Calcineurin, Bcl-w OSTN	―
Heart diseases	MEF2C MEF2D	Ca^2+^signaling pathway	Tdgf1,Suv39h1	Atorvastatin
Muscle disorders	MEF2A MEF2C MEF2D	PI3K/AKT pathway p38 MAPK pathway	BDNF,KLF6 RUFY3, GSK3β	―
Cancer	MEF2B MEF2C MEF2D	Wnt signaling pathway	VEGF,SFRP2 HDAC4	Oleanolic acid

It is noteworthy that the MEF2 proteins could be potential targets during the treatment of many diseases. Although previous studies have reported various functions of MEF2 family proteins, further works are still needed to clarify the pathological and pharmacological mechanism by which MEF2 genes is connected to specific signaling pathway. The detailed elucidation of underlying mechanisms of MEF2 activation and its downstream targets could facilitate the development of efficient MEF2-targeted therapeutic strategies.
